# Responsiveness of the “PROM Pediatric Physical Therapy” in Dutch Pediatric Physical Therapy Practices

**DOI:** 10.1097/PEP.0000000000001250

**Published:** 2025-12-30

**Authors:** Lieze P.G. Hoogveld, Philip J. van der Wees, Reinier P. Akkermans, Anjo J.W.M. Janssen

**Affiliations:** Radboud University Medical Center, Amalia Children’s Hospital, Department of Rehabilitation, Pediatric Physical Therapy, Nijmegen, the Netherlands (Ms Hoogveld and Dr Janssen); Radboud University Medical Center, Department of Rehabilitation, Nijmegen, the Netherlands (Dr van der Wees); Radboud University Medical Center, IQ Health Science Department, Nijmegen, the Netherlands, (Dr van der Wees and Mr Akkermans).

**Keywords:** child, measurement properties, motor, patient-reported outcome measure, pediatric physical therapy, responsiveness

## Abstract

**Purpose:**

To investigate the responsiveness of the patient-reported outcome measure pediatric physical therapy (PROM-ppt), a questionnaire used to also stimulate shared decision making in Dutch pediatric physical therapy practices.

**Methods:**

Children completed the PROM-ppt at intake and 3 months after intervention or post-intervention. Reported problems were categorized as motor or pain related goals for intervention. Responsiveness was examined to test the a priori hypotheses and area under the curve (AUC). The Global Perceived Effect scale was used for comparison.

**Results:**

Overall, the hypotheses were confirmed in 60% of the cases with adequate AUCs. In children with motor problems, 80% of the hypotheses were matched, with adequate AUCs. In children with pain related problems, 40% of the hypotheses were matched, with inadequate AUCs.

**Conclusion:**

The PROM-ppt had moderate responsiveness in the pediatric population, good responsiveness in children with interventions for motor problems, and poor responsiveness to pain related problems.

**WHAT THIS ADDS TO THE EVIDENCE**

**Current evidence**: Repeated assessments using standardized, discriminative, and norm-referenced motor tests by pediatric physical therapists (PPTs) are common and necessary to assess motor performance in children with motor problems and to evaluate the effects of interventions.

**Gap in the evidence**: Motor tests are not sufficiently sensitive to detect changes in specific motor skills and activities over time.^2,3^ This limits the monitoring and evaluation of personalized interventions. Patient-reported outcome measures (PROMs) can contribute to the evaluation of intervention effects at the activity level.^4^ However, the existing PROMs for adults are unsuitable for children and their specific motor problems.^8^

**How did this study fill this evidence gap?** This study assessed the responsiveness of a specifically developed PROM for pediatric physical therapy (PROM-ppt).^12^ The PROM-ppt focuses on motor activities in which children experience difficulties. Problems in performing these activities form the basis for intervention goals in PPT treatment, which are formulated through shared decision making among the PPT, child, and parents. PPTs can use the PROM-ppt to evaluate the effectiveness of personalized interventions from the perspective of the child and parents. Based on these results, the PROM-ppt appears to be a responsive instrument for measuring changes over time, especially in children with motor problems.

**Implication of all the evidence to clinicians**: The PROM-ppt is a responsive instrument for motor problems. Its use is recommended to assess the child’s perspective on agreed goals for intervention in shared decision making. PPTs can use the PROM-ppt to monitor intervention effects from the child’s or parent’s perspective in pediatric physical therapy practice.

## INTRODUCTION

Pediatric physical therapists (PPTs) provide personalized interventions for children with motor problems^1^ often using standardized, discriminative, and norm-referenced motor tests to classify motor performance and monitor and evaluate intervention effects.^1^ These motor tests have good to excellent validity and high reliability.^1^ However, both intra- and inter-individual variabilities in motor performance are typical developmental characteristics of children.^2^,^3^ Moreover, intra-individual variability in motor development is nonlinear.^3^ This means that children develop in their unique ways in leaps and bounds. In some cases, children may have higher scores using reference norms at one assessment, and score much lower in the next, or vice versa. This makes longitudinal evaluation of motor performance difficult. Additionally, motor tests are often insufficiently sensitive to detect changes in motor skills trained during interventions of PPTs.^2^,^3^ Therefore, monitoring and evaluating the effects of personalized interventions is challenging.

Patient-reported outcome measures (PROMs) are questionnaires that offer a complementary approach for evaluating intervention effects, from the patients’ perspective and are increasingly used as indicators of the quality of care.^4^-^7^ While PROMs are frequently applied in adult care (30-95% usage rates), their use in children is limited, as existing questionnaires were not specifically designed for children or their specific motor problems.^8^-^11^ Therefore, at the request of the Dutch Association of Pediatric Physical Therapists, a PROM for pediatric physical therapy (PROM-ppt) was developed, with the additional goal of improving shared decision making.^12^

This PROM-ppt focuses on motor activities in which children experience difficulties. Problems in performing these activities form the basis of intervention goals in PPT treatment. Involving children in decisions about their care is important.^13^ Given the complexity and variability of motor development, most parents and children are unlikely to accurately estimate achievable goals themselves. The PROM-ppt supports shared decision-making, ensuring realistic, prioritized goals, set together. Thus, this PROM-ppt helps PPTs evaluate the effectiveness of personalized interventions from the perspective of children and parents. By combining motor tests with the PROM-ppt, PPTs can integrate objective assessments with patient-centered care, and the patient’s perspective, for a more comprehensive evaluation of intervention effects. Nevertheless, the measurement properties of PROM-ppt must be established before it can be implemented in daily practice. The first version of the PROM-ppt had excellent test-retest reliability and adequate content validity.^12^ In addition, the responsiveness must be known to ensure that changes measured are interpreted correctly.

This study aimed to determine the responsiveness of the PROM-ppt in children aged 0-18 years in Dutch physical therapy practices using a construct approach with hypothesis testing and a criterion approach, with the Global Perceived Effect (GPE) scale as the gold standard.^14^,^15^

## METHODS

This pre-post study conducted in Dutch physical therapy practices, followed the Consensus-based Standards for the Selection of Health Status Measurement Instrument (COSMIN) and Standards for Reporting Diagnostic Accuracy Studies (STARD) reporting guidelines.^16^,^17^ The study was approved by the Medical Ethics Committee of our institution (registration number: 2019-5800). Written informed consent was obtained from parents and children.

### Participants

The PPTs were recruited between September 2021 and May 2023 by sending email invitations to physical therapy practices in all Dutch regions, distributing flyers at a conference for PPTs, and placing invitations in the Dutch Pediatric Physical Therapy Newsletter. Between October 2021 and May 2023, the PPTs enrolled newly referred children aged 0-18 years with motor difficulties and indications for PPT intervention by convenience sampling. Children (and their parents) with insufficient Dutch language skills were excluded.

The study followed the COSMIN guidance for sample size for responsiveness.^14^,^18^ For the criterium approach, a sample size of 30-50 patients is considered adequate for the smallest group. Regarding the construct approach, a sample size of 50-99 patients is considered adequate. We aimed to obtain a total sample size of 100 participants.

### Measurements

#### Patient-reported outcome measure in pediatric physical therapy (PROM-ppt)

The PROM-ppt was developed using a RAND-modified Delphi procedure to reach a consensus on the activities included and the scoring of the PROM-ppt because a systematic literature search for PROMs in 2015 did not yield a suitable questionnaire (506 questionnaires were reviewed).^8^,^12^

The PROM-ppt focuses on specific motor activities in which children experience difficulties and assesses intervention goals related to these activities (Supplemental Digital Content 1, available at: http://links.lww.com/PPT/A658). It is paper-based and includes a section with categorized activities to be checked by the child or their parents at the initial appointment. The categories are: “gross motor activities,” “fine motor activities,” “sports and play,” “activities of daily living,” and “ball skills.” After assessment by the PPT, together selected activities are prioritized by the child and PPT to obtain the top 3-5, and intervention goals are set at the activity level. In the scoring section of the PROM, selected activities are specified and scored by the child or the parent(s) on the “Ability,” “Satisfaction,” and “Pain” components, each on a numerical rating scale from 0 to 10. The “Ability” component, in which the child is asked about their performance level during the activity, has a scale ranging from “0” for “cannot perform” to “10” representing “can perform well.” The “Satisfaction” component, in which the child is asked about their satisfaction with the activity, ranges from “0” for “not satisfied” to “10” for “very satisfied.” The “Pain” component, in which the child is asked about the level of pain while performing the activity, ranges from “0” for “no pain” to “10” for “worst pain imaginable,” and the scoring aligns with the numeric pain rating scale.^19^,^20^ After a period of intervention or at the end, the intervention goals are assessed again to evaluate the “Ability,” “Satisfaction,” and “Pain” components. Parents of children under 8 years of age may complete the PROM-ppt for their children, children between 8 and 16 years of age may complete the PROM-ppt with their parents and adolescents over 16 years of age can complete the PROM-ppt themselves.

The test-retest reliability of the PROM-ppt is excellent^.^^12^ The content validity has been established through the developmental process and expert evaluation so that it accurately reflects motor activities relevant to PPT. The responsiveness of the PROM-ppt was studied in 2018 and was considered adequate.^12^ In retrospect, the method was suboptimal because only a criterion approach was used, and no hypotheses were tested according to a construct approach. Moreover, in 2020, a pilot study was conducted to assess the feasibility and initial experiences of children, parents, and PPTs with the PROM-ppt in several practices. Qualitative studies were conducted,^21^ and the PROM-ppt was adapted and simplified.

The PROM-ppt component “Pain” is an 11-point numerical rating scale (NRS-11). The NRS-11 is a valid and responsive tool for assessing pain intensity in children as young as 6 years.^19^,^20^

#### Global Perceived Effect scale

The GPE is an instrument that assesses patients` perception of the effectiveness of and satisfaction with the intervention. The GPE questions in this study were reworded for use in children and defined as follows: Question 1 (Q1): “Rate your ability to perform the skills relative to the start of the intervention” and Question 2 (Q2): “How satisfied are you with the intervention?”. Responses were scored on a 7-point Likert scale as follows. For Q1: 1 = “very much improved,” 2 = “much improved,” 3 = “slightly improved,” 4 = “no change,” 5 = “slightly worsened,” 6 = “much worsened,” and 7 = “vastly worsened.” For Q2: 1 = “completely satisfied,” 2 = “very satisfied,” 3 = “somewhat satisfied,” 4 = “neither satisfied nor dissatisfied, 5 = “somewhat dissatisfied,” 6 = “very dissatisfied,” and 7 = “absolutely dissatisfied.” The GPE has demonstrated adequate reliability, validity, and responsiveness in patients with chronic low back pain.^22^,^23^

### Procedure

Participating PPTs received instructions on how to use the PROM-ppt via a PowerPoint video, handout, and information materials (paper PROM-ppt and GPE forms, information letters for children and parents, and informed consent forms). The participating PPTs held an online introductory meeting with the first author and an evaluation meeting after 2 months.

During the child’s initial PPT intake, a minimum of 3 and a maximum of 5 intervention goals were jointly identified. Subsequently, the children or their parent(s) were asked to complete the PROM-ppt (*t*_0_). The intervention effect was assessed at the end of the intervention or after 3 months. The child or parent(s) were asked to score the PROM-ppt (2nd scoring section) and GPE (*t*_1_). The GPE was used as a comparison instrument to assess the child’s or parent’s perceptions of the effectiveness of the intervention.

### Statistical analysis

Descriptive statistics, numbers, percentages, means, and standard deviations were used to assess the childrens’ characteristics. Normality was assessed and normally distributed data were reported with mean and standard deviation.

Spearman’s rank correlation coefficient was used to determine the correlations between PROM-ppt change and GPE scores. This enabled the testing of a priori constructed hypotheses. The area under the curve (AUC) of the receiver operating characteristic (ROC) curve was calculated as a measure of the PROM-ppt’s ability to discriminate between improvement and no improvement during the intervention.^24^,^25^

To determine the change scores of the PROM-ppt for each child, we first calculated the change scores by subtracting the scores at t_0_ from the score at *t*_1_ (*t*_1_-*t*_0_) for each component of all 3 to 5 selected activities, divided by the number of selected activities.

#### Construct approach

For hypotheses testing, we calculated the mean change score for all 3 combined components, referred to as the PROM-ppt total change score. The PROM-ppt total change score was calculated for all selected activities (Table 1, Hypothesis 4) and for the activity prioritized as number 1 (Table 1, Hypothesis 5). We combined the 3 components of the PROM-ppt to test the hypotheses because we hypothesized that these components jointly contribute to a child’s overall experience of performing an activity. For this, the component “Pain” was recoded (that a higher score indicated less pain) to enable a comparison of scores; that is, higher scores indicated better results. Data were excluded from the analysis if a score was missing in the PROM-ppt components or if the GPE ratings were missing.

**TABLE 1:** A Priori Hypotheses to Assess the Responsiveness of the PROM Pediatric Physical Therapy (PROM-ppt) with the Global Perceived Effect Scale (GPE) as Comparator Instrument

	Comparison	Expected Spearman correlation strength (direction)	Explanation
1) Change score PROM-ppt component “Ability”	GPE Q1	0.5-0.8 (+)	The PROM-ppt component ‘Ability’ and GPE question 1 both ask about changes in the ability to perform activities. We expect a moderate to good correlation^a^
2) Change score PROM-ppt component “Satisfaction”	GPE Q1	0.4-0.7 (+)	GPE question 1 asks about the ability to perform activities. PROM-ppt asks about satisfaction with this performance. The constructs are different; however, they influence each other. Therefore, we expect a fair to moderate correlation^a^
3) Change score PROM-ppt component “Satisfaction”	GPE Q2	0.4-0.7 (+)	The PROM-ppt component ‘Satisfaction’ and GPE question 2 both ask about satisfaction. PROM-ppt asks about satisfaction with the performance of activities, and GPE question 2 asks about satisfaction with the intervention. The constructs are different but influence each other. We expect a fair to moderate correlation^a^
4) Total change score PROM-ppt	GPE Q1	0.4-0.7 (+)	The GPE and the PROM-ppt both include questions about ability. The GPE asks about changes in the ability to perform activities. PROM-ppt asks about ability, satisfaction and pain separately for each activity. We expect these change scores to vary by skill. We expect a fair to moderate correlation^a^
5) Change score PROM-ppt activity 1	GPE Q1	0.5-0.8 (+)	GPE asks in 1 question, about changes in the ability to perform activities. We expect the PROM-ppt change scores to vary by activity. We expect that the activity prioritized as number 1, which the child has the most difficulty with or would most like to improve first, will have the greatest impact on the GPE score. Therefore we expect a moderate to good correlation^a^

Abbreviations: SD, standard deviation; Q, question; PROM, Patient reported outcome measure.

^a^ Strength of the Spearman’s correlation coefficients: little or no relationship ≤0.25; low to fair 0.25 to 0.50; moderate to good 0.50 to 0.75; strong relationship ≥0.75.^26^^.^

Five hypotheses were constructed (Table 1) regarding the expected correlation between the change scores of the PROM-ppt components and GPE scores. The strength of the Spearman’s correlation coefficients is interpreted as follows: little or no relationship, ≤0.25; low to fair, 0.25-0.50; moderate to good, 0.50-0.75; strong relationship, ≥0.75.^26^ Responsiveness was considered good if more than 75% of the hypotheses were confirmed, moderate if 50-75% were confirmed, and poor if less than 50% were confirmed.^15^,^26^

#### Criterion approach

The GPE score was dichotomized, and children were identified as “improved” (for GPE Q1) or “satisfied” (for GPE Q2) if they had a GPE score of 1 or 2 and were identified as “not improved” or “not satisfied” if they had a GPE score ≥ 3.^14^ The PROM-ppt component “Ability” was considered a similar construct to the construct measured by the GPE’s Q1: “Rate the ability to perform skills relative to the start of the intervention.” The PROM-ppt component “Satisfaction” was considered a similar construct to the construct measured by the GPE’s Q2: “How satisfied are you with the intervention?”. For 2 PROM-ppt components, “Ability” and “Satisfaction” ROC curves were constructed for 3 groups: the total group, for children with motor problems, and for children with pain-related problems. Cut-off points represent the calculated sensitivity and 1-specificity based on the proportion of the change score of the PROM-ppts of children identified as “improved” and “satisfied” (GPE score 1 or 2) or “not improved” and “not satisfied” (GPE ≥3).^14^ The AUC was calculated, and AUCs ≥0.70 were considered adequate.^14^

## RESULTS

**Fig. 1.**
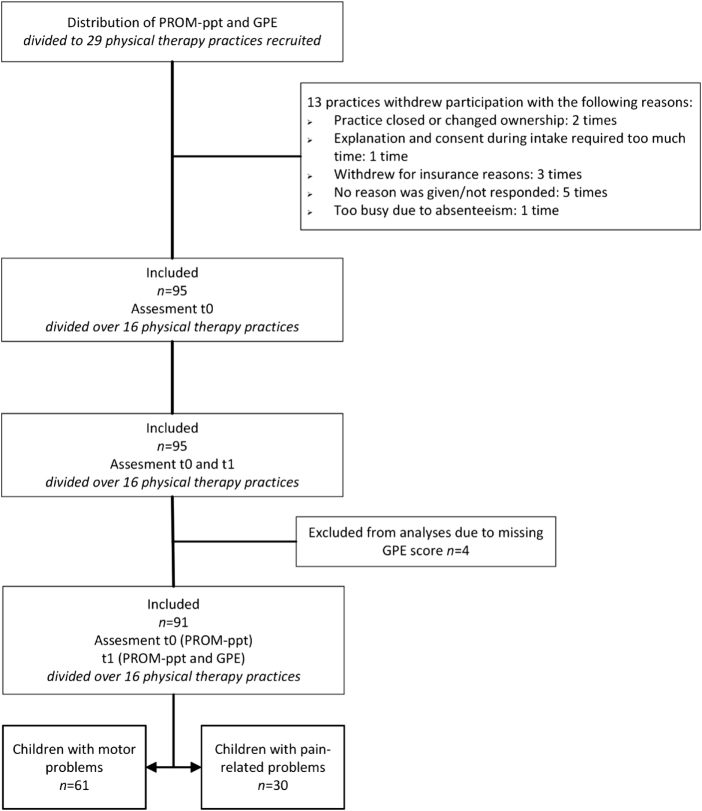
Flow chart for PROM pediatric physical therapy (PROM-ppt) distributed and included. Abbreviations: GPE, Global Perceived Effect scale; PROM, Patient-Reported Outcome Measures; PROM-ppt, PROM Pediatric Physical Therapy.

The PROM-ppt was distributed across 29 practices (Figure 1) between October 2021 and May 2023. Sixteen physical therapy practices in 6 regions of the Netherlands participated in this study. In total, 95 children participated, and 4 were excluded from the analyses owing to missing GPE scores, resulting in the inclusion of 91 children. The group consisted of 46 (48%) girls aged 0-17.5 years, with a mean age of 8 years 2 months (SD, 4 years and 6 months). Table 2 has the characteristics of the total group and the subgroups with motor problems (67%) and pain-related problems (34%).

**TABLE 2:** Characteristics of Children in the Total Group and the Groups With Motor Problems and Pain-Related Problems

Characteristic	Total group *n* = 91	Motor problems *n* = 61	Pain-related problems *n* = 30
Girls (%)	46 (48)	22 (36)	24 (73)
Age (years)			
Mean (SD)	8.13 (4.51)	6.26 (4.06)	11.86 (2.68)
Range	0.1-17.5	0.1-17.1	6.5-17.5
Skill categories chosen by children *n* (%)			
Gross motor activities	189 (57)	117 (53)	72 (65)
Fine motor activities	52 (16)	51 (23)	1 (1)
Sports and games	40 (12)	14 (6)	26 (23)
Activities of daily living	37 (11)	27 (12)	10 (9)
Ball skills	15 (5)	13 (6)	2 (2)
Total number of sessions			
Mean (SD)	8.72 (4.53)	9.21 (4.88)	7.88 (3.82)
Range	1–23	1–23	3–18
Intervention duration (days), mean (SD)	83.07 (34.11)	92.02 (36.12)	67.32 (23.51)

Abbreviation: SD, standard deviation.

The GPE Q1 score classified 65 (71%) children as improved and 26 (29%) as unimproved in their ability to perform the selected and trained motor activities. Supplemental Digital Content 2 (available at: http://links.lww.com/PPT/A659) presents details of the PROM-ppt change scores (mean and SD) and changes in the GPE.

Overall, 87 children (95.6%) reported satisfaction with their PPT intervention in the GPE (Supplemental Digital Content 2, available at: http://links.lww.com/PPT/A659). Of these, 35 (38.5%) were completely satisfied, 52 (57.1%) were very satisfied, and 4 (4.4%) were somewhat satisfied.

### Responsiveness

#### Construct approach

The Spearman correlations ranged from 0.21 to 0.59 (Table 3). For the total group (*n* = 91), 3 of 5 correlations (60%) between PROM-ppt change scores and GPE scores were consistent with our hypotheses (Table 3). For the group with motor problems (*n* = 61), 4 of 5 correlations (80%) between the change scores of the PROM-ppt and GPE scores were consistent with our hypotheses. For children with pain-related problems (*n* = 30), 2 of 5 correlations (40%) between the change scores of the PROM-ppt and the GPE were in agreement with our hypotheses.

**TABLE 3:** Spearman Correlations (*r*_s_) of Change Scores PROM Pediatric Physical Therapy (PROM-ppt) and Global Perceived Effect Scale (GPE) for the Total Group and Groups with Motor Problems and Pain-related Problems

	Comparison		Total group *n* = 91		Motor problems *n* = 61		Pain-related problems *n* = 30	
	GPE	Hypothesis	Spearman correlation, 95% CI, p-value	Hypotheses met (yes/no)	Spearman correlation, 95% CI, p-value	Hypotheses met (yes/no)	Spearman correlation, 95% CI, p-value	Hypotheses met (yes/no)
Change score PROM-ppt component “Ability”	Q1	1	0.43, 0.24-0.59, p<.001	no	0.52, 0.30-0.69, p<.001	yes	0.25, -0.13-0.57, p.186	no
Change score PROM-ppt component “Satisfaction”	Q1	2	0.50, 0.33-0.65, p<.001	yes	0.55, 0.33-0.71, p<.001	yes	0.35, -0.03-0.64, p.059	no
	Q2	3	0.33 0.13-0.51 p.001	no	0.21, -0.06-0.44 p.113	no	0.51, 0.17-0.74 p.004	yes
Total change score PROM-ppt	Q1	4	0.56, 0.39-0.69, p<.001	yes	0.56, 0.36-0.72, p<.001	yes	0.51, 0.16-0.73, p.005	yes
Total change score PROM-ppt activity 1	Q1	5	0.53, 0.31-0.63, p<.001	yes	0.55, 0.34-0.71 p<.001	yes	0.34, -0.05-0.62, p.074	no
Number of accepted hypotheses (%)				3 (60%)		4 (80%)		2 (40%)

Abbreviation: Q, question; CI, confidence interval.

Strength of the Spearman’s correlation coefficients: little or no relationship ≤0.25; low to fair 0.25 to 0.50; moderate to good 0.50 to 0.75; strong relationship ≥0.75.^26^^.^

#### Criterion approach

**Fig. 2.**
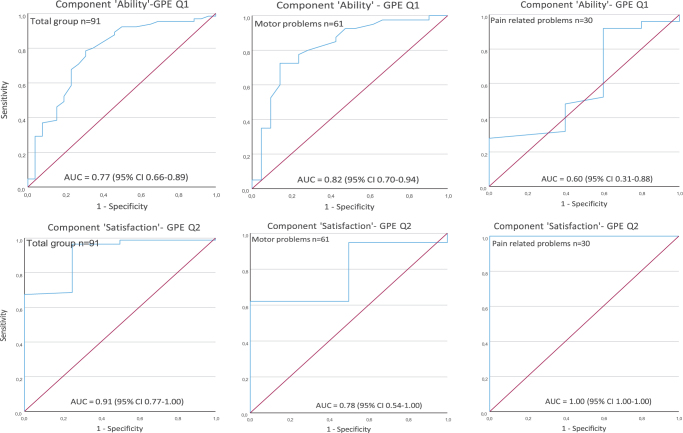
Receiver operating characteristics curves of the change score on the PROM pediatric physical therapy (PROM-ppt) components “Ability” and “Satisfaction” for the total group, children with motor problems, and children with pain-related problems. Abbreviations: ROC, receiver operating characteristic; PROM, Patient-Reported Outcome Measures; PROM-ppt, PROM pediatric physical therapy; GPE, Global Perceived Effect Scale; Q1, question 1; AUC, area under the curve.

AUCs were calculated for the total group, for children with motor and pain-related problems. Figure 2 graphs the ROC curves of the PROM-ppt change scores for the “Ability” and “Satisfaction” components. The AUC of PROM-ppt component “Ability,” according to GPE Q1, was 0.77 (95% CI: 0.66-0.89) for the total group, 0.82 (95% CI: 0.70-0.94) for children with motor problems and 0.60 (95% CI: 0.31-0.88) for children with pain-related problems. The AUC of PROM-ppt component “Satisfaction,” according to GPE Q2, was 0.91 (95% CI: 0.77-1.00) for the total group, 0.78 (95% CI: 0.54-1.00) for children with motor problems and 1.00 (95% CI: 1.00-1.00) for children with pain-related problems.

## DISCUSSION

Using a construct approach, we found that the PROM-ppt is moderately responsive for the total group of children, confirming 60% of hypotheses when comparing changes in PROM-ppt scores with perceived improvements in the GPE. For the subgroup of children with motor problems, the PROM-ppt demonstrated a good responsiveness, confirming 80% of the hypotheses. However, we could not confirm this hypothesis in the subgroup of children with pain-related problems.

The criterion approach had responsiveness for the total group and for children with motor problems with adequate AUCs, indicating the ability of the PROM-ppt to discriminate between children who improved and those who did not during the PPT intervention. Similar to the construct approach, limited responsiveness was observed in children with pain-related problems. Thus, PROM-ppt can best be used in children with motor problems.

When compared with other outcome measures, the PROM-ppt partly aligns with the Patient-Specific Functional Scale (PSFS), Goal Attainment Scaling (GAS), and the Canadian Occupational Performance Measure (COPM), all demonstrated adequate responsiveness.^28^,^29^ However, neither GAS nor PSFS has been studied in children, limiting direct comparisons.

The COPM, used and studied in pediatric populations, takes about 30 minutes to administer. The interview is more comprehensive, making it suitable for complex cases and long-term rehabilitation goals, just like GAS. This comprehensive and time-consuming nature limits its feasibility in primary PPT practices. In addition, the PROM-ppt focuses directly on motor activities in which children experience difficulties, and on intervention goals related to these activities. However, both tools, include shared decision-making and the perspectives of the child and parents.^29^

The PROM-ppt components “Ability” and “Satisfaction” were adequate responsive, as previously investigated by Overvelde et al.^12^ The change scores of the PROM-ppt component “Ability” exhibited a fair relationship with the GPE Q1 “Rate the ability to perform the skills relative to the start of the intervention” (*r*_s_ = 0.43) for the total group. As hypothesized, a moderate-to-good relationship (*r*_s_ = 0.52) of the “Ability” component with GPE Q1 was found for children with motor problems but little or no relationship (*r*_s_ = 0.25) for pain-related problems. This relationship for children with motor problems seems logical because the goals of the chosen activities are directly related to motor performance. For example, if a child aims to improve their ability to bounce a ball, their perceived competence will correspond to the improvement experienced compared to baseline. This alignment between activity goals and motor skills explains the strong responsiveness of the PROM-ppt, as it reflects changes in targeted motor abilities.

The AUCs of the “Ability” component according to GPE Q1 were adequate and confirmed the responsiveness for the total group and children with motor problems. However, for children with pain-related problems, the PROM-ppt component “Ability” was less strongly correlated. Pain is a multidimensional problem and transformed into an activity level, reflects a broader perception than just the experience of pain. This was reflected in the scores of the “Ability” component, which appeared more erratic in these children. The observed effects may have been amplified by the relatively small sample size of children with pain-related problems. In some cases, there were large negative change scores on the “Ability” component if pain increased during the treatment (the activity could then, for example, no longer be performed at a second measurement). In other cases, PROM-ppt change scores were relatively low on the “Ability” component, whereas a high score (better performance to the start of the intervention) was given on GPE Q1 owing to strong pain reduction. Consistent with this, the AUC of the “Ability” component, according to the GPE Q1 for children with pain-related problems, was inadequate.

Among children with motor problems, our findings revealed little to no relationship between the PROM-ppt component “Satisfaction” (about the performance of the activities) and GPE Q2 (satisfaction with the intervention). Overall, 96.7% of the children with motor problems reported being satisfied with the intervention, regardless of their satisfaction with the performance of the activities. This is encouraging for Dutch PPTs; however, it mostly indicates that children value interventions for their motor problems, regardless of the treatment effects. Satisfaction with activities and treatment appear to be 2 different constructs. Conversely, the PROM-ppt component “Satisfaction” was responsive in children with pain-related problems. The results had a moderate to good relationship with GPE Q2, “How satisfied are you with the intervention?” (*r*_s_ = 0.51). In contrast to children with motor problems, satisfaction with the intervention (GPE Q2) among children with pain-related problems was related to their ability to perform the selected activities. For children experiencing pain during motor activities, satisfaction with an intervention appears to depend primarily on its effectiveness. When the treatment was less effective, the children were less satisfied, and vice versa. Moreover, parental reports on pain, especially for younger children, may limit accuracy, as parents do not directly experience the pain.^27^ This may also explain our low responsiveness in the pain-related group, though smaller sample sizes limit the conclusions we can draw.

The AUCs of the “Satisfaction” component, according to GPE Q2, were adequate for all 3 groups (total, motor-, and pain-related).

We hypothesized a fair to moderate relationship between the “Satisfaction” component and GPE Q1 (ability to perform activities relative to the start of the intervention). Although the constructs differ, they are expected to influence each other. In the total group and in children with motor problems, results had a moderate-to-good relationship (*r*_s_ = 0.50, *r*_s_ = 0.55) with GPE Q1. However, only a fair relationship was observed in children with pain-related problems (*r*_s_ = 0.35). In this group, the PROM-ppt scores for component “Satisfaction” also had erratic scores, with large negative change scores for the “Satisfaction” component when pain increased and relatively low change scores when pain decreased during intervention (while high scores, ie, better performance compared to the start of the intervention, were given on GPE Q1).

The PROM-ppt outcome might be influenced by the number of sessions, which we examined in an exploratory analysis. We found a significant but low to moderate negative association, showing a tendency for higher satisfaction and ability to perform activities to be reported after fewer sessions.

Video Abstract, Supplemental Digital Content 3 (available at: http://links.lww.com/PPT/A660) for more insights from the authors.

### Strengths and limitations

A strength of this study is the use of both criterion and construct approaches, with a priori hypotheses for expected correlations between the change scores of the PROM-ppt and the comparison instrument, following the COSMIN recommendations.

However, limitations must be considered interpreting our findings in children with pain-related problems. The recommended sample size of at least 50 was not met for this group.

Furthermore, the use of the GPE as a comparator instrument is a limitation. Kamper et al. found that GPE ratings are strongly influenced by the current status of patients and questioned whether they truly reflect changes, particularly when measured over several months, as in our study.^22^ However, a more appropriate instrument for distinguishing between improved and unimproved motor skills in children was unavailable.

While the GPE has been used in several studies, the validity of the GPE when used in children remains unclear. This highlights the need for a responsive tool to measure changes in children. We hypothesize that the responsiveness of the PROM-ppt might be higher than that of the GPE when used in children.

A potential limitation is the lack of comparisons with therapist-assessed measures like Goal Attainment Scaling (GAS), which future research could explore to complement the PROM-ppt.

### Clinical implications

The PROM-ppt can be used in clinical practice in the Dutch situation for children with motor-related problems to evaluate PPT treatment outcomes; however, it is not responsive for evaluating treatment outcomes in children with pain-related problems.

### Implications for future research

Future research should focus on criterion validity to strengthen the PROM-ppt’s robustness and clinical utility. Determining the minimal important change for different groups of children is crucial, this represents the threshold value of the change considered important by the child or parent.^30^ PPTs can then interpret the PROM-ppt change scores to monitor intervention effects from the child’s or parent’s perspectives.
